# An Innovative Approach to Informing Research: Gathering Perspectives on Diabetes Care Challenges From an Online Patient Community

**DOI:** 10.2196/ijmr.3856

**Published:** 2015-06-30

**Authors:** Emily B Schroeder, Jay Desai, Julie A Schmittdiel, Andrea R Paolino, Jennifer L Schneider, Glenn K Goodrich, Jean M Lawrence, Katherine M Newton, Gregory A Nichols, Patrick J O'Connor, Marcy Fitz-Randolph, John F Steiner

**Affiliations:** ^1^ Institute for Health Research Kaiser Permanente Colorado Denver, CO United States; ^2^ Department of Medicine University of Colorado Denver Aurora, CO United States; ^3^ HealthPartners Institute for Education and Research Minneapolis, MN United States; ^4^ Division of Research Kaiser Permanente Northern California Oakland, CA United States; ^5^ Kaiser Permanente Center for Health Research Portland, OR United States; ^6^ Department of Research & Evaluation Kaiser Permanente Southern California Pasadena, CA United States; ^7^ Group Health Research Institute Seattle, WA United States; ^8^ PatientsLikeMe Cambridge, MA United States

**Keywords:** social networking, diabetes mellitus, quality of health care, patient centered outcome research

## Abstract

**Background:**

Funding agencies and researchers increasingly recognize the importance of patient stakeholder engagement in research. Despite calls for greater patient engagement, few studies have engaged a broad-based online community of patient stakeholders in the early stages of the research development process.

**Objective:**

The objective of our study was to inform a research priority-setting agenda by using a Web-based survey to gather perceptions of important and difficult aspects of diabetes care from patient members of a social networking site-based community.

**Methods:**

Invitations to participate in a Web-based survey were sent by email to members of the PatientsLikeMe online diabetes community. The survey asked both quantitative and qualitative questions addressing individuals’ level of difficulty with diabetes care, provider communication, medication management, diet and exercise, and relationships with others. Qualitative responses were analyzed using content analysis.

**Results:**

Of 6219 PatientsLikeMe members with diabetes who were sent survey invitations, 1044 (16.79%) opened the invitation and 320 (5.15% of 6219; 30.65% of 1044) completed the survey within 23 days. Of the 320 respondents, 33 (10.3%) reported having Type 1 diabetes; 107 (33.4%), Type 2 diabetes and taking insulin; and 180 (56.3%), Type 2 diabetes and taking oral agents or controlling their diabetes with lifestyle modifications. Compared to 2005-2010 National Health and Nutrition Examination Survey data for individuals with diabetes, our respondents were younger (mean age 55.8 years, SD 9.9 vs 59.4 years, SE 0.5); less likely to be male (111/320, 34.6% vs 48.4%); and less likely to be a racial or ethnic minority (40/312, 12.8% vs 37.5%). Of 29 potential challenges in diabetes care, 19 were categorized as difficult by 20% or more of respondents. Both quantitative and qualitative results indicated that top patient challenges were lifestyle concerns (diet, physical activity, weight, and stress) and interpersonal concerns (trying not to be a burden to others, getting support from family/friends). In our quantitative analysis, similar concerns were expressed across patient subgroups.

**Conclusions:**

Lifestyle and interpersonal factors were particularly challenging for our online sample of adults with Type 1 or Type 2 diabetes. Our study demonstrates the innovative use of social networking sites and online communities to gather rapid, meaningful, and relevant patient perspectives that can be used to inform the development of research agendas.

## Introduction

Funding agencies and researchers have increasingly recognized the importance of incorporating patient stakeholders into all phases of the research process [1,2]. Patient stakeholder engagement can take place at various points in the “life cycle” of research, including question formulation, study design, interpretation of results, and dissemination of findings [1,3]. The best way to engage patient stakeholders in the research process has not been well defined, and likely depends on the topic under study, the patient population concerned, and the stage of the research process [1,3]. Patient stakeholder engagement can take various forms. For example, patients may act as participants in individual interviews, focus groups, or in-person stakeholder meetings; or as research team collaborators; or may become involved through social networking sites (SNSs) [1,3,4]. In diabetes research, for example, only a few previous studies have described patients’ research priorities and preferences [5-7]. These studies gathered data via focus groups in a single community [6], a questionnaire printed in a German weekly news magazine [5], and a multi-step process including online and paper forms of a survey distributed by a variety of Type 1 diabetes advocacy organizations [7]. Due to time restrictions and resources, most methods of patient stakeholder engagement are limited to using small, highly selective samples [8].

SNSs, which have been growing in popularity, can engage large numbers of thoughtful patient stakeholders about a range of health and research topics, and can do so more quickly and at lower cost than traditional survey methods [8,9]. PatientsLikeMe is one such health- and disease-related SNS that has more than 190,000 members internationally. It offers patients tools to track their illness, share health data with peers, and participate in research studies [10]. PatientsLikeMe has established a level of trust among its users, which is an important component of stakeholder engagement [8,11]. Researchers using PatientsLikeMe have published more than 30 peer-reviewed publications [12] focusing on conditions such as amyotrophic lateral sclerosis [13-15], Parkinson’s disease [10,13,16], multiple sclerosis [17], epilepsy [18], mood disorders [13], and organ transplantation [19].

The SUPREME-DM (SUrveillance, PREvention, and ManagEment of Diabetes Mellitus) network is a consortium of 11 member organizations of the HMO Research Network [20,21]. Research institutes embedded in these health systems have developed the largest and most clinically-detailed cohort of privately-insured patients with diabetes in the United States (the *DataLink*) [22]. The SUPREME-2 (SUPREME-DM: Sustaining a Learning Research Network) study was funded by the Agency for Healthcare Research and Quality in 2013, with the goal of enhancing the capacity of the SUPREME-DM DataLink to address the research priorities of patient stakeholders [23]. Over a 12-month period, we used a systems-based participatory research approach to engage stakeholders. This included a one-day stakeholder meeting held in Washington, DC in 2014, followed by a webinar virtual meeting [23,24]. Stakeholders included patients, researchers, and leaders of health care delivery systems, federal agencies, membership/advocacy groups, and disadvantaged populations. However, we wanted to ensure that the voices of diabetes patients figured prominently in our stakeholder engagement process. Given time and resource constraints, the PatientsLikeMe online diabetes community provided an opportunity to engage with a geographically-diverse, thoughtful, and interested group of individuals with diabetes. This population rapidly provided qualitative and quantitative patient perspectives that informed and supplemented the research preferences and priorities that were identified during the in-person and virtual stakeholder engagement processes.

The overall purpose of our online survey was to obtain research preferences and priorities from an interested group of individuals with diabetes that could be used to help guide subsequent SUPREME-DM stakeholder engagement and research priorities. Our specific research aims were (1) to identify issues in diabetes management that PatientsLikeMe patient stakeholders find difficult or important in the following domains: accessing diabetes care, communication with providers, medication management, lifestyle behaviors, and personal relationships; (2) to gather an array of patient perspectives that would inform, amplify, and supplement the findings from the in-person stakeholder meeting; and (3) to assess the pragmatic usefulness of online surveys for conducting diabetes research among an SNS population.

## Methods

### The SUPREME-DM Network

The SUPREME-DM network was funded by the Agency for Healthcare Research and Quality (R01HS019859) in 2010 [20,21]. SUPREME-DM is a consortium of 11 US integrated health systems. Research institutes embedded within these health systems have developed a distributed virtual data warehouse that contains the following types of anonymous de-identified information gathered from their electronic health record and administrative data systems: demographic characteristics, outpatient pharmacy dispensing details, laboratory tests and associated results, and diagnosis and procedure codes from outpatient and inpatient health care encounters [25]. The resulting diabetes DataLink has been used in research on diabetes surveillance and comparative effectiveness, and includes information collected from 2005 onward for approximately 16 million adults and 2.5 million children in 10 states [21]. A subsequent study, the SUPREME-2 study, was funded by the Agency for Healthcare Research and Quality (1R01HS022963-01) in 2013, with the goal of enhancing the capacity of the SUPREME-DM DataLink to address the research priorities of patient and organizational stakeholders [23].

### Survey Design and Implementation

To rapidly obtain input from patient stakeholders, we conducted an online survey with the PatientsLikeMe diabetes community (see Multimedia Appendix 1). Because individuals in the PatientsLikeMe network have volunteered to share personal information for surveys such as ours, and because we did not collect any identifying information, this survey was not considered human subjects research by the Kaiser Permanente Colorado Institutional Review Board. No incentives were provided to participants.

The survey was developed by SUPREME-DM investigators in collaboration with PatientsLikeMe staff. The survey was pilot tested by 50 PatientsLikeMe members, and modified based on their responses. The final survey is provided in Multimedia Appendix 1. The survey included several questions modified from the National Health and Nutrition Examination Surveys (NHANES) and the Behavioral Risk Factor Surveillance Surveys (BRFSS). The modified questions were related to age, race, ethnicity, gender, type of diabetes, duration of diabetes, general health status, primary activity, types of medication, diabetes complications, weight, and height [26,27]. The remainder of the survey used 29 items to assess patient experience with and challenges in diabetes care, provider communication, medication management, and other topics (eg, lifestyle behaviors and personal relationships). Each topic domain was preceded by the prompt, “We want to hear which questions about [domain heading] are most important to you right now. Please tell us which of these concerns about diabetes impacts you personally, and how difficult each one makes your life.” The five response options were: not difficult, a little difficult, somewhat difficult, very difficult, and does not apply. There were also three global, open-ended questions,: (1) “Are there any other challenges or concerns about your diabetes care that you want us to know?”, (2) “Thinking about when you were first told you had diabetes, what was or would have been most helpful for you to know about your diabetes at that time?”, and (3) “Thinking about 3-5 years into the future from now, what do you feel will be important to learn or know about your diabetes? Why?”

Using the PatientsLikeMe *Private Message* tool, we sent invitations to the individual email accounts of eligible PatientLikeMe members from the diabetes community. The invitation encouraged those participants to log in to their PatientsLikeMe account and complete the Web-based survey. Inclusion criteria were: self-reported Type 1 or Type 2 diabetes, aged 18 years or older, resident of the United States, and email contact from PatientsLikeMe Private Messages enabled. We also required participants to be active participants in PatientsLikeMe. We defined ‘active’ as any activity during the past 360 days for those with Type 1 diabetes, and 3 or more log-ins during the past 90 days for those with Type 2 diabetes. We sent the invitation to individuals with unknown ages or locations of residence, but if they indicated on their survey that they were younger than 18 years or lived outside the United States, then their responses were discarded. The survey was open for 23 days (2014 Jan 15 through 2014 Feb 6), and reminder emails were sent at 3 days and 14 days after the initial invitation. Participants could complete the survey over several sessions if they desired.

### Analysis

If participants did not reach the end of the survey, then their responses were not analyzed. We calculated the mean and standard deviation for continuous variables and percentage for categorical variables. Body mass index (BMI) was categorized as normal (BMI < 25.0 kg/m^2^), overweight (BMI = 25.0-29.9 kg/m^2^), Class I obese (BMI = 30.0-34.9 kg/m^2^), and Class II obese or higher (BMI ≥ 35.0 kg/m^2^). To report measures of patient experience, we combined the percentages of individuals responding “somewhat difficult” or “very difficult”, and removed from the denominator individuals who indicated “does not apply.” We compared results by diabetes type and insulin status (Type 1 diabetes, Type 2 diabetes taking insulin, and Type 2 diabetes not taking insulin); gender; age; general health status; depression; and primary activity. For significance testing, we used two-sided Fisher Exact tests for categorical variables and Kruskal-Wallace tests for continuous variables. However, as this was an exploratory analysis of a convenience sample, we did not primarily rely upon statistical significance testing, but instead looked for magnitude of the response, meaningful differences in percentages, and/or relative rankings.

To examine the three qualitative, open-ended measures, we used a content analysis approach to provide an overview of the major themes emerging from each question [28-30]. In an effort to keep the open-ended comments grounded in the experiences of respondents, analysis was conducted by an objective investigator (JLS) with expertise in qualitative analyses who was not aware of the closed-ended survey results. An initial reading of open-ended responses to understand the general participant response and context to each specific question was followed by a second reading to summarize content. For example, if a participant wrote “I wished a doctor had told me to take diabetes more seriously earlier on…,” then this was summarized as “take more seriously.” Upon a third reading of the open-ended responses, summary phrases were condensed into themes, but with enough detail to preserve their intended meaning. We then tabulated the frequencies with which these themes were expressed. When possible, themes were aggregated into summary themes. Responses could reflect a single or multiple themes. The themes were then shared with the study team for comment and consensus. In this paper, we provide the list of these themes and the frequency of text responses for each theme mentioned.

## Results

### Respondent Characteristics

Of the 6219 PatientsLikeMe members with diabetes who were invited to participate, 1044 (16.79%) opened the invitation. Of those who opened the invitation, 188 opted out, 273 did not start the survey, 194 started but did not complete the survey, and 389 completed the survey. Of the 389 who completed the survey, 69 were excluded because they resided outside of the United States, leaving a final sample size of 320 (5.15% of 6219; 30.65% of 1044). Compared to the 5899 who were not included in the analysis, the 320 complete responders were older (mean age 55.8 years, SD 9.9 vs 53.7 years, SD 11.3, *P*<.001); more likely to have Type 1 diabetes (33/320, 10.3% vs 419/5899, 7.10%, *P*=.04); and possibly more likely to be female (209/320, 65.3% vs 3563/5899, 60.40%, *P*=.09).

Of the 320 respondents, 33 (10.3%) had Type 1 diabetes, 107 (33.4%) had Type 2 diabetes and were taking insulin, and 180 (56.3%) had Type 2 diabetes and were on oral agents (but not insulin) or controlled their diabetes with lifestyle interventions alone. Individuals with Type 1 diabetes were younger than those with Type 2 diabetes, had a lower BMI, longer duration of diabetes, were more likely to be employed, and reported better overall general health than individuals with Type 2 diabetes (Table 1). Rates of self-reported diabetes complications and comorbidities were high, and except for kidney disease and retinopathy, tended to be higher among individuals with Type 2 diabetes than among individuals with Type 1 diabetes (Table 1). Compared to the NHANES 2005-2010 diabetes population, our sample had a higher percentage of females, more non-Hispanic whites, and a smaller proportion of individuals aged 65 years or older [31] (Table 1).

**Table 1:** Characteristics of PatientsLikeMe survey respondents (n=320).

		Full cohort	Type 1 diabetes	Type 2 diabetes taking insulin	Type 2 diabetes not taking insulin	*P* value^a^	NHANES^b^ 2005-2010 [31]
		n (%) or mean (SD)	n (%) or mean (SD)	n (%) or mean (SD)	n (%) or mean (SD)		
Female, n (%)		209/320 (65.3)	22/33 (66.7)	65/107 (60.8)	122/180 (67.8)	.49	51.6
**Race/ethnicity, n (%)**							
	Non-Hispanic white	272/312 (87.2)	28/32 (87.5)	88/102 (82.4)	160/178 (89.9)	.14	62.5
	Non-Hispanic black	17/312 (5.5)	0/32 (0.0)	9/102 (8.8)	8/178 (4.5)		16.7
	Other	23/312 (7.4)	4/32 (12.5)	9/102 (8.8)	10/178 (5.6)		20.8
**Age, n (%) or years, mean (SD)**							
	Years, mean (SD)	55.8 (9.9)	47.7 (12.9)	57.1 (9.0)	56.5 (9.1)	<.001	59.4
	< 55 years, n (%)	130/320 (40.6)	21/33 (63.6)	37/107 (34.6)	72/180 (40.0)	.03	60.5
	55-64 years, n (%)	136/320 (42.5)	11/33 (33.3)	50/107 (46.7)	75/180 (41.7)		
	> 65 years, n (%)	54/320 (16.9)	1/33 (3.0)	20/107 (18.7)	33/180 (18.3)		39.5
**Body mass index, n (%) or kg/m** ^**2**^ **, mean (SD)**							
	kg/m^2^, mean (SD)	33.9 (8.3)	26.9 (5.0)	36.2 (8.4)	33.9 (8.1)	<.001	32.8
	< 25.0 kg/m^2^, n (%)	45/318 (14.2)	16/33 (48.5)	8/105 (7.6)	21/180 (11.7)	<.001	12.9
	25.0-29.9 kg/m^2^, n (%)	66/318 (20.8)	6/33 (18.2)	19/105 (18.1)	41/180 (22.8)		25.9
	30.0-34.9 kg/m^2^, n (%)	80/318 (25.2)	8/33 (24.2)	20/105 (19.1)	52/180 (28.9)		61.2
	≥35.0 kg/m^2^, n (%)	127/318 (39.9)	3/33 (9.1)	58/105 (55.2)	66/180 (36.7)		
**Duration of diabetes, n (%) or years, mean (SD)**							
	Years, mean (SD)	12.1 (11.4)	28.0 (15.2)	12.9 (9.2)	8.7 (9.0)	<.001	
	0-5 years, n (%)	108/320 (33.8)	3/33 (9.1)	22/107 (20.6)	83/180 (46.1)	<.001	
	5-10 years, n (%)	78/320 (24.4)	4/33 (12.1)	31/107 (29.0)	43/180 (23.9)		
	> 10 years, n (%)	134/320 (41.9)	26/33 (78.8)	54/107 (50.5)	54/180 (30.0)		
**Education, n (%)**							
	Some high school	1/319 (0.3)	0/33 (0.0)	0/106 (0.0)	1/180 (0.6)	.26	
	High school graduate	56/319 (17.6)	3/33 (9.1)	19/106 (17.9)	34/180 (18.9)		
	Some college	136/319 (42.6)	10/33 (30.3)	43/106 (40.6)	83/180 (46.1)		
	College graduate	81/319 (25.4)	12/33 (36.4)	28/106 (26.4)	41/180 (22.8)		
	Post graduate	45/319 (14.1)	8/33 (24.2)	16/106 (15.1)	21/180 (11.7)		
**Primary activity, n (%)**							
	Employed	109/320 (34.1)	19/33 (57.6)	25/107 (23.4)	65/180 (36.1)	.001	
	Homemaker	16/320 (5.0)	3/33 (9.1)	4/107 (3.7)	9/180 (5.0)		
	Student	5/320 (1.6)	1/33 (3.0)	3/107 (2.8)	1/180 (0.6)		
	Out of work	17/320 (5.3)	3/33 (9.1)	8/107 (7.5)	6/180 (3.3)		
	Unable to work	91/320 (28.4)	5/33 (15.2)	37/107 (34.6)	49/180 (27.2)		
	Retired	82/320 (25.6)	2/33 (6.1)	30/107 (28.0)	50/180 (27.8)		
**General health, n (%)**							
	Excellent	7/318 (2.2)	2/33 (6.1)	1/106 (0.9)	4/179 (2.2)	.007	
	Very good	48/318 (15.1)	12/33 (36.4)	10/106 (9.4)	26/179 (14.5)		
	Good	109/318 (34.3)	6/33 (18.2)	35/106 (33.0)	68/179 (38.0)		
	Fair	94/318 (29.6)	9/33 (27.3)	33/106 (31.1)	52/179 (29.1)		
	Poor	60/318 (18.9)	4/33 (12.1)	27/106 (25.5)	29/179 (16.2)		
**Complications, n (%)**							
	High blood pressure	227/318 (71.4)	15/33 (45.5)	88/106 (83.0)	124/179 (69.3)	<.001	
	High cholesterol	243/312 (77.9)	17/32 (53.1)	86/104 (82.7)	140/176 (79.6)	.003	
	Depression	189/315 (60.0)	18/33 (54.6)	64/106 (60.4)	107/176 (60.8)	.79	
	Heart attack	27/315 (8.6)	0/33 (0.0)	18/105 (17.1)	9/177 (5.1)	<.001	
	Heart disease	50/315 (15.9)	6/33 (18.2)	27/105 (25.7)	17/177 (9.6)	.001	
	Stroke	22/312 (7.1)	2/33 (6.1)	10/103 (9.7)	10/176 (5.7)	.46	
	Kidney disease	28/310 (9.0)	4/33 (12.1)	15/101 (14.9)	9/176 (5.1)	.02	
	Retinopathy	66/305 (21.6)	12/31 (38.7)	33/104 (31.7)	21/170 (12.4)	<.001	
	Neuropathy	148/304 (48.7)	13/33 (39.4)	67/103 (65.1)	68/168 (40.5)	<.001	
	Extremity ulcers	49/313 (15.7)	5/33 (15.2)	22/103 (21.4)	22/177 (12.4)	.14	

^a^A 3-way comparison of Type 1 diabetes, Type 2 diabetes taking insulin, and Type 2 diabetes not taking insulin.

^b^National Health and Nutrition Examination Surveys.

### Quantitative Findings

Table 2 shows the topics that were found “somewhat difficult” or “very difficult” by 20% or more of respondents. The full survey, with all 29 topics, is found in Multimedia Appendix 1. Respondents most often identified difficulties in the Diet and Exercise domain, with between 50.5% and 66.1% citing all four topics in this domain as difficult. All topics in the Relationships With Others domain were also found difficult by 25.3%-38.0% of respondents. Individuals also indicated high levels of difficulty with “paying for my diabetes visits, treatment, or supplies;” “seeing specialty providers such as endocrinologists, diabetes educators, dieticians, etc;” “testing my blood sugars;” “managing side effects of interactions between my medications;” “using alternative medicine;” and a number of the topics within the Communication domain.

In subgroup analysis, individuals with Type 1 diabetes generally reported lower levels of difficulty than did individuals with Type 2 diabetes (see Multimedia Appendix 2). Exceptions (where the percentage of individuals with Type 1 diabetes reporting difficulty with a topic was at least 5% higher than that of individuals with Type 2 diabetes) included some topics in the Getting Diabetes Care and Communication domains, as well as “managing side effects of interactions between my medications,” and “trying not to be a burden to others.” Individuals with Type 2 diabetes who were taking insulin nearly uniformly reported higher levels of difficulty for all domains than those with Type 2 diabetes who were treated only with oral agents or lifestyle modification. Diet and Exercise topics were identified as most difficult for both groups, and both groups mainly ranked these topics similarly (see Multimedia Appendix 2).

Subgroup analyses by gender, age (< 55 years vs >55 years), and duration of diabetes showed minimal differences (data not shown). Women were more likely than men to report difficulty with “feeling that my doctor respects, understands, and listens to me” (52/206, 25.2% vs 17/108, 15.7%), “testing my blood sugars” (64/201, 31.8% vs 21/107, 19.6%), “getting enough physical activity” (151/208, 72.6% vs 60/111, 54.1%), “managing my weight” (152/208, 73.1% vs 52/111, 46.8%), and “managing stress” (129/207, 62.3% vs 49/110, 44.6%). Frequencies of difficulty in most domains were greater among individuals reporting fair or poor health compared to those reporting excellent, very good, or good health; individuals with depression compared to those without depression; and individuals whose primary activity was “unable to work” or “other” compared to those whose primary activity was “retired” or “employed” (data not shown).

**Table 2:** Survey domains for which at least 20% of participants responded “very difficult” or “difficult” (n=320).

Domain	Topic	n (%)
**Getting diabetes care**		
	Paying for my diabetes visits, treatment, or supplies	106/307 (34.5)
	Seeing specialty providers such as endocrinologists, diabetes educators, dieticians, etc	83/291 (28.5)
**Communication**		
	Using e-mail, texting, or the Web to reach my health care provider	68/231 (29.4)
	Making sure that all my diabetes care providers are working together for me	77/298 (25.8)
	Making choices about diabetes medicine and other treatments that I think are best for me	76/301 (25.2)
	Feeling that my doctor or other health care providers respect, understand, and listen to me	69/314 (22.0)
	Working with my doctor to set personal goals for my treatment	66/309 (21.4)
**Medications**		
	Testing my blood sugars	85/308 (27.6)
	Managing side effects of interactions between my medications	76/275 (27.6)
	Using alternative medicine (natural herbs, acupuncture, meditation, etc)	43/163 (26.4)
**Diet and exercise**		
	Getting enough physical activity	211/319 (66.1)
	Managing my weight	204/319 (64.0)
	Managing stress	178/317 (56.2)
	Eating a healthy diet	161/319 (50.5)
**Relationships with others**		
	Trying not to be a burden to others	112/295 (38.0)
	Getting enough support from my family and friends	117/317 (37.0)
	Diabetes interfering with my work	51/194 (26.3)
	Diabetes interfering with my social activities with family, friends, neighbors, or groups	71/281 (25.3)

### Qualitative Findings

The open-ended survey questions elicited numerous responses (259-299 responses to each of the three questions). Summary themes for 2 of the 3 questions are shown in Tables 3 and 4, and illustrative quotes for all three questions are shown in Multimedia Appendices 3-5. Summary themes included: educational needs (more instruction about healthy lifestyle behaviors, diabetes as a disease, diabetes care management, and taking medications); the need for shared decision-making and compassionate support; care access concerns (such as referral issues and costs); participants’ desires to know more about disease progression, new treatments and technologies, long-term care and lifestyle management options, and possibilities for reversing or curing diabetes; and the challenges of managing comorbid conditions (such as depression, rheumatoid arthritis, fibromyalgia, and heart disease).

**Table 3:** Summary themes for responses to the question, “Thinking about when you were first told you had diabetes, what was or would have been most helpful for you to know about your diabetes at that time?” (n=299).^a^

Summary theme	Theme	n
**Lifestyle education**		90
	Education in diet, carbohydrate consumption, and food preparation	49
	Clear, realistic assessment of lifestyle goals to work on (diet, exercise, smoking, etc)	16
	Education and support in weight loss (ie, gyms, classes, bariatric surgery)	15
	Education on importance of active lifestyle and ongoing exercise routine	7
	Encouragement to avoid stress and stay calm and relaxed	3
**Shared decision making, compassion, reassurance, and support**		87
	Realistic discussion with provider about the seriousness of diabetes, that it requires diligent and daily self-care and treatment, and that it can be managed	28
	At time of diagnosis, support in dealing with denial or overwhelming fear of diabetes	16
	Hope and reassurance that a person with diabetes progression can be slowed, possibly reversed, and that a person can live a normal and good life	16
	Open, two-way discussion with provider on all aspects of diabetes and treatment options	13
	Credible, helpful resources: information on diabetes (books, web, literature) and also social support groups	10
	Compassion from provider with no blaming or shaming of person trying to manage their diabetes	4
**Education on the disease of diabetes**		86
	Education in diabetes progression, long-term consequences, side effects, and symptoms of worsening	39
	Education on what diabetes is, its causes, and contributing factors	20
	Clear explanation of blood sugars, A1c levels, and insulin resistance	8
	Education on diabetes interactions and impacts on comorbid conditions (depression, hypothyroidism, kidney disease, hypoglycemia)	9
	Awareness and education about transitioning from prediabetes to diabetes	6
	Awareness and education about pregnancy and diabetes, including gestational diabetes	4
**Diabetes care management education**		36
	Education on the importance and reason for routinely self-monitoring blood glucose levels	13
	Stronger emphasis/education that diabetes can be managed and treated well with diet, lifestyle, and medications	23
**Care access, referrals, and costs**		24
	Timely access/referrals to specialists (ie, endocrinologists), dieticians, and diabetes educators/classes at time of diagnosis and as part of ongoing care	17
	Timely access to medications and durable equipment (i.e. insulin pump)	4
	Realistic appraisal of immediate and long-term diabetes financial impact (ie, provider costs, medications, blood glucose monitors, test strips)	3
**Education about diabetes medications**		23
	Clear explanations about medication side-effects (eg, digestive, impotence, weight gain) and alternative medication options	10
	Understanding of how medications are processed by the body, including the time at which they are taken	8
	Education of non-pharmaceutical alternatives or providers	5

^a^Because an individual’s response could reflect multiple themes, the total number of n values for the themes is greater than the total number of respondents (n=299). The n value for a summary theme is the sum of the n values for the individual themes within that category.

**Table 4:** Summary themes for responses to the question, “Thinking about 3-5 years into the future from now, what do you feel will be important to learn or know about your diabetes? Why?” (n=283).^a^

Summary theme	Theme	n
**Understand disease progression**		120
	Progression and impact on overall health and aging	60
	Progression and impact on specific organ systems (ie, eyes, kidneys, heart, depression, neuropathy, etc)	60
**Long-term control, treatment, and care management strategies**		106
	How to effectively maintain and manage blood sugars over the years	54
	How to avoid or best manage medication use, especially insulin use	19
	How to manage neuropathy, its associated pain, and prevent amputations	15
	How to have an ongoing individualized, personalized, and integrated care management plan	10
	How to continue to care for diabetes and know as much as possible about it over the years	8
**Awareness of new treatments and technologies**		72
	Current science, research, and technology for new ways to treat and manage diabetes	36
	Information on: new medications, new glucose management technologies, improved diet/lifestyle options	36
**Long-term lifestyle management strategies**		43
	Knowing more about healthy eating, nutrition, and access to proper foods over the years	32
	Having an exercise routine tailored to a person’s lifestyle and physical limitations as years progress	11
**Managing future costs**		40
	How to manage and afford ongoing costs of medications, test supplies, foods, exercise fees, etc	20
	How to manage and afford any increase in costs of medications, test supplies, foods, exercise fees, etc	20
**Reverse or cure diabetes**		37
	Strongly desire a cure be found	28
	Desire to know how to reverse diabetes, including use of bariatric surgery	9
**Managing overall health and well-being**		23
	How to live longer, healthier, happier, and better with diabetes	16
	How to accept living with diabetes and the need for lifestyle changes	7

^a^Because an individual’s response could reflect multiple themes, the total number of n values for the themes is greater than the total number of respondents (n=283). The n value for a summary theme is the sum of the n values for the individual themes within that category.

## Discussion

### Principal Findings

Our findings illustrate that administering a survey using SNSs and online communities is feasible, and can rapidly generate rich sets of quantitative and qualitative data that may be used to inform research priorities. In this study, we reached over 1000 potential participants with diabetes and completed our data collection in just 23 days.

Over the past two decades, many studies have explored the underlying barriers to and facilitators of diabetes care delivery, self-management, and education from the patient’s perspective [32-38]. Our findings generally agree with previous studies, and provide some assurance that SNSs and their associated online communities do generate important insights about patients’ needs that may inform future research. At the same time, these communities offer a setting that facilitates the efficient and rapid collection of patient input and perspectives. The open-ended responses analyzed in this study provided a particularly large and rich set of qualitative data that compare favorably to the types of information generally obtained through in-person focus groups or individual interviews.

We felt it was important to collect information about patients’ concerns for the future, as this is the perspective that motivates many research agendas. We acknowledge that individuals generally have difficulty predicting preferences for future states, and thus, that participants may have had problems accurately addressing this question [39]. However, since our population included people along the diabetes continuum (from recently diagnosed diabetes to long standing diabetes with multiple complications), we believe that we were able to adequately capture individuals’ perspectives throughout the diabetes life cycle.

Our survey was motivated by the desire to inform the development of an ongoing research agenda for the SUPREME-DM network. Only a few previous studies have described patients’ research priorities and preferences related to diabetes, and these were limited in scope and/or participation [5-7]. One study used a questionnaire printed in a German weekly news magazine in order to prioritize nine broad research topics (“development, pathophysiology and prevention of diabetes,” “transplantation and cell therapy,” etc) [5]. Another study described a series of six focus groups in a single community that involved a total of 39 adults with diabetes [6]. A third study used a multi-step process that included online and paper forms of a survey distributed by diabetes advocacy organizations, but its focus was limited to research priorities for Type 1 diabetes [7]. In choosing the best method to establish patients' research priorities and preferences, which is a crucial task in patient engagement, researchers need to weigh a variety of competing interests that include timeframe, budget, size and representativeness of the patient sample, and desired depth of responses (qualitative vs quantitative) [4,40]. The benefits of our SNS-based approach included short timeframe, large patient sample, and depth of responses.

Well-recognized limitations of online surveys based on SNSs include selection bias due to the potential unrepresentativeness of the members of these communities, and low response rates [17]. In addition, items such as age, weight, type of diabetes, and comorbidities are self-reported, which is a problem shared with other online surveys and population-based surveys such as BRFSS and components of NHANES.

Compared to the 2005-2010 NHANES sample of individuals with self-reported diabetes, our sample differed in a number of important demographic characteristics [31]. This raises concerns that the difficulties expressed by our sample may not be representative of those most prevalent within the broader diabetes community. Given that, in our sample, the levels of self-reported comorbidities (including depression) were high and self-reported overall health status was low, we suspect that the sample included an overrepresentation of individuals with more severe diabetes [41]. Previous research in other PatientsLikeMe communities has found that the PatientsLikeMe population tends to have a higher proportion of females [19], non-Hispanic whites [19], more educated individuals [17], and younger adults [16,17], relative to an external comparator population with the same disease condition. We confirmed these findings by comparing our data to 2005-2010 NHANES data for individuals with diabetes.

We received complete responses from only 5.15% (320/6219) of individuals who were invited to participate. Part of this stems from the difficulty of identifying active users of the SNS. Only 16.79% (1044/6219) of individuals who were sent invitations to participate in the survey actually opened them. Of those who opened the invitation, we received complete responses from 37.26% (389/1044), of whom we subsequently excluded 6.61% (69/1044) who did not meet the eligibility criterion of US residence, for a final response rate of 30.65% (320/1044). Expected response rates for these types of online survey have not been well described, but were between 9% and 36% for previous PatientsLikeMe surveys [42-44]. Individuals responding to our survey represent those who are most active on an online SNS, and therefore are different from the general population with diabetes in many important ways, including level of engagement in their health care, access to technology, available time to participate in these communities, and level of education. Thus, our findings cannot be extrapolated to the broader population of individuals with diabetes.

Despite these limitations, the approach to patient engagement we describe here had a number of strengths [17]. First, it was easy to identify individuals who were interested and engaged in participating. Second, we were able to collect information from a relatively large number of patient stakeholders in very little time (23 days of survey availability, and a total of 93 days from initial planning to survey close, including 14 days of planned downtime due to holidays). Third, we were able to collect a wealth of qualitative data, only a small sample of which has been included in this manuscript. Fourth, this was a very cost-effective approach. Fifth, participants expressed gratitude for being given the opportunities to participate in research that was important to them and to share their stories and feedback via the open-ended measures. One potential verification strategy would be to circulate the results back to the original survey sample and ask for agreement or disagreement with these, or for further interpretation of the data. We did subsequently present these results to patient stakeholders at an in-person meeting, and found that those patients’ diabetes care experiences resonated well with those articulated by PatientsLikeMe respondents.

### Conclusions

Lifestyle and interpersonal factors were of particular concern to our online sample of adults with diabetes and, overall, difficulties associated with care for the condition were similar for adults with Type 1 or Type 2 diabetes. Our experience shows that SNSs and online communities can be accessed to quickly gain meaningful and relevant patient perspectives that can help inform the development of a research agenda.

## Appendix

Multimedia Appendix 1 PatientsLikeMe online diabetes survey.

Multimedia Appendix 2 Percentage of participants responding “very difficult” or “difficult” for each domain, by type of diabetes and insulin status (n=320).

Multimedia Appendix 3 Summary of responses to the question, “Thinking about when you were first told you had diabetes, what was or would have been most helpful for you to know about your diabetes at that time?” (n=299).

Multimedia Appendix 4 Summary of responses to the question, “Thinking about 3-5 years into the future from now, what do you feel will be important to learn or know about your diabetes? Why?” (n=283).

Multimedia Appendix 5 Summary of responses to the question, “Are there any other challenges about your diabetes care that you want us to know?” (n=257).

## Abbreviations

BMI: body mass index
BRFSS: Behavioral Risk Factor Surveillance Surveys
NHANES: National Health and Nutrition Examination Surveys
SNS: social networking site
SUPREME-DM: SUrveillance, PREvention, and ManagEment of Diabetes Mellitus
