# Supplementary material for: An Innovative Approach to Informing Research: Gathering Perspectives on Diabetes Care Challenges From an Online Patient Community
Source: Interact J Med Res. 2015 Jun 30;4(2):e13. doi: 10.2196/ijmr.3856 (PMC4526969; doi:10.2196/ijmr.3856)
Supplement: Multimedia Appendix 1 [file ijmr_v4i2e13_app1.pdf]

Multimedia Appendix 1: PatientsLikeMe online diabetes survey.

Section	
**Private Message Invitation**	**Diabetes Research - What is important to you?**<br><br>Hi USERNAME,<br><br>This one's a little bit different. This is us and our new partner in this project Kaiser Permanente learning more about diabetes from you – in your own words. We'll still ask about a few basics, but we're also going to leave some questions open ended so you can add in your own comments. Tell us what you find difficult about diabetes treatment and care, and what you think is important for researchers to look at down the road.<br><br>Click below to get started on this new survey. We're listening.<br>Thanks for sharing!<br><br>PatientsLikeMe and Kaiser Permanente
**Introduction**	**Thanks for participating in this survey. It should take between 15 and 20 minutes to complete. And don't forget, there are a bunch of questions with comment boxes where you can add any additional answers or thoughts. You can come back and finish the survey at any time by clicking on your invitation message.**<br><br>**Your voice will help everyone learn more about diabetes treatment and care. You'll also help doctors and researchers understand what areas of diabetes research matter most to you. As always with our research, we'll be sure to share the results with the community. Let's get started.**
**BasicInfo (Demographics)**	**Sex**<br>Female<br>Male
	**Birth date**<br>Year-Month-Day
	**Country**<br>Drop down list or free text if not on list
	**Ethnicity**<br>Hispanic or Latino<br>Not Hispanic or Latino
	**Race**<br>American Indian or Alaskan Native<br>Asian<br>Black or African American<br>Mixed Race<br>Native Hawaiian or other Pacific Islander<br>White<br>I prefer not to answer
	**Education Level**<br>8th grade or less (left school around 14)<br>Some high school, but did not graduate (left school around 16)

	High school graduate or GED (left school around 18)<br>Some college but less than a bachelor's / undergraduate degree<br>College bachelor's / undergraduate degree<br>Postgraduate degree (Master's, doctorate, etc.)<br>I prefer not to answer
**Section 1 (Background information)**	**Would you say your general health is:**<br>Excellent<br>Very good<br>Good<br>Fair<br>Poor<br>Don't know/Not sure
	**What type of diabetes do you have?**<br>Type 1<br>Type 2<br>Other<br>Not sure<br><br>*Show if answer="Other"*<br>**You said you had some other type of diabetes. What type of diabetes do you have?**<br>Free-form text
	**What is your primary activity?**<br>Employed for wages<br>Self-employed<br>Out of work for one year or more<br>Out of work for less than one year<br>Homemaker<br>Student<br>Retired<br>Unable to work
	**How old were you when a doctor, nurse, or other health professional told you (or your parents) that you have diabetes? If you don't know your exact age, just make your best guess.**
	**Are you currently taking any medications to treat your diabetes?**<br>Yes<br>No<br>Don't know<br><br>*Show if answer(medications)=Yes*<br>**Are you currently taking insulin?**<br>Yes<br>No<br>Don't know<br><br>*Show if answer (insulin)=Yes*<br>**How old were you when you first started taking insulin at least once a day? If you do not know your exact age, just make your best guess.**<br>Number

	Show if answer (medications)=Yes<br>**Are you currently taking medications (other than insulin) to lower your blood sugar?**<br>Yes<br>No<br>Don't know
	**Have you ever been told by a doctor, nurse, or other health professional that you have any of the conditions described below?**<br>• **High blood pressure?**<br>• **High blood cholesterol?**<br>• **A depressive disorder, including depression, major depression, dysthymia, or minor depression?**<br>• **A heart attack, also called a myocardial infarction?**<br>• **Angina or coronary heart disease?**<br>• **A stroke?**<br>• **Kidney disease because of your diabetes?**<br>• **Diabetes has affected your eyes or that you had retinopathy?**<br>• **Diabetes has affected your nerves/nervous system or that you have neuropathy?**<br>Yes<br>No<br>Don't know
	**Have your ever had any sores or irritations on your feet that took more than four weeks to heal?**<br>Yes<br>No<br>Don't know
**Section 2**	**We want to hear which questions about your diabetes care are most important to you right now. Please tell us which of these concerns about diabetes impacts you personally, and how difficult each one makes your life. You can give the same answer for more than one item.**
	**What do you personally find difficult about getting the diabetes care that you need?**<br>• **Having a regular doctor or other health care provider for my diabetes.**<br>• **Seeing specialty providers such as endocrinologists, diabetes educators, dieticians, etc.**<br>• **Getting to the office of my doctor or other health care provider.**<br>• **Getting an appointment at a doctor's office or clinic as soon as I think one is needed.**<br>• **Getting or keeping health insurance coverage.**<br>• **Paying for my diabetes visits, treatment or supplies.**<br>Not difficult<br>A little difficult<br>Somewhat difficult<br>Very difficult<br>Does not apply
	**Please tell us more about what you personally find difficult about getting the diabetes care you need.**<br>Free-form text

**Section 3**	**What do you personally find difficult about communicating with your doctors or other health care providers? You can give the same answer for more than one item.**<br>• **Getting a response from my doctor or other health care professionals in a timely manner.**<br>• **Using e-mail, texting or the Web to reach my doctor or other health care provider.**<br>• **Making choices about diabetes medicine and other treatments that I think are best for me.**<br>• **Talking with my doctor or health care provider about the pros and cons of each choice for my treatment.**<br>• **Working with my doctor to set personal goals for my treatment.**<br>• **Feeling that my doctor or other health care providers respect, understand, and listen to me.**<br>• **Getting easy to understand instructions about taking care of my diabetes.**<br>• **Understanding current and future health risks of my diabetes, such as its effects on my heart, eyes, kidneys, feet, or mental health.**<br>• **Making sure that all my diabetes care providers are working together for me.**<br>Not difficult<br>A little difficult<br>Somewhat difficult<br>Very difficult<br>Does not apply
	**Please tell us more about what you personally find difficult about communicating with your doctors or other health care providers.**<br>Free-form text
**Section 4**	**What do you personally find difficult about managing your medications for diabetes and other conditions? You can give the same answer for more than one item.**<br>• **Taking insulin.**<br>• **Taking diabetes medications (other than insulin) as prescribed.**<br>• **Taking medications for cholesterol or blood pressure as prescribed.**<br>• **Managing side effects or interactions between my medications.**<br>Not difficult<br>A little difficult<br>Somewhat difficult<br>Very difficult<br>Does not apply
	**Please tell us more about what you personally find difficult about managing your medications for diabetes.**<br>Free-form text
**Section 5**	**What other things do you find difficult about taking care of your diabetes and other conditions? You can give the same answer for more than one item.**<br>• **Testing my blood sugars ("fingersticks" or continuous glucose monitoring).**<br>• **Eating a healthy diet.**

	• **Getting enough physical activity.**<br>• **Managing my weight.**<br>• **Managing stress.**<br>• **Getting enough support from my family and friends.**<br>• **Diabetes interfering with my work.**<br>• **Diabetes interfering with my social activities with family, friends, neighbors or groups.**<br>• **Trying to not be a burden to others.**<br>• **Using alternative medicine (natural herbs, acupuncture, meditation, etc).**<br>Not difficult<br>A little difficult<br>Somewhat difficult<br>Very difficult<br>Does not apply
	**Please tell us more about what you personally find difficult about taking care of your diabetes and other conditions.**<br>Free-form text
**Section 6**	**How important are the following things to you?**<br>• **Feeling as well as possible.**<br>• **Living as long as possible.**<br>• **Being able to do as many activities as possible.**<br>Not important<br>A little important<br>Somewhat important<br>Very important
**Section 7**	**Are there any other challenges or concerns about your diabetes care that you want us to know?**<br>Free-form text
	**Thinking about when you were first told you had diabetes, what was or would have been most helpful for you to know about your diabetes at that time?**<br>Free-form text
	**Thinking about 3-5 years into the future from now, what do you feel will be important to learn or know about your diabetes? Why?**<br>Free-form text
**Section 8**	**Final questions! Please tell us just a little more about you.**<br><br>**About how much do you weigh (in pounds) without shoes?**<br>Number
	**About how tall are you without shoes?**<br>(options 3'6" through 7'6")
	**Do you have any last comments about this survey?**<br>Free-form text
