# Supplementary material for: An Innovative Approach to Informing Research: Gathering Perspectives on Diabetes Care Challenges From an Online Patient Community
Source: Interact J Med Res. 2015 Jun 30;4(2):e13. doi: 10.2196/ijmr.3856 (PMC4526969; doi:10.2196/ijmr.3856)
Supplement: Multimedia Appendix 2 [file ijmr_v4i2e13_app2.pdf]

Multimedia Appendix Table 1. Percent of Participants Responding "Very Difficult" or "Difficult" for Each Domain, by Type of Diabetes and Insulin Status, N=320.

	Full Cohort N=320		Type 1 Diabetes N=33		Type 2 Diabetes N=287		Type 2 Diabetes Taking Insulin N=107		Type 2 Diabetes Not Taking Insulin N=180	
	%	Rank[a]	%	Rank	%	Rank	%	Rank	%	Rank
**Getting Diabetes Care**										
Paying for my diabetes visits, treatment or supplies	106/307(34.5)	7	10/32 (31.3)	7	96/275 (34.9)	7	45/103 (43.7)	6	51/172 (29.7)	9
Seeing specialty providers such as endocrinologists, diabetes educators, dieticians, etc	83/291 (28.5)	9	3/32 (9.4)	24 (tie[a])	80/259 (30.9)	8	32/104 (30.8)	14	48/155 (31.0)	6
Getting or keeping health insurance coverage	52/308 (16.9)	22	4/32 (12.5)	23	48/276 (17.4)	23	20/102 (19.6)	23	28/174 (16.1)	22
Getting an appointment at a doctor's office or clinic as soon as I think one is needed	51/315 (16.2)	24	8/32 (25.0)	10	43/283 (15.2)	25	18/106 (17.0)	25	25/177 (14.1)	23
Having a regular health care provider for my diabetes	50/314 (15.9)	25	7/33 (21.2)	16 (tie)	43/281 (15.3)	24	21/106 (19.8)	22	22/175 (12.6)	25
Getting to the office of my health care provider	39/314 (12.4)	27	3/32 (9.4)	24 (tie)	36/282 (12.8)	26	17/107 (15.9)	26	19/175 (10.9)	26
**Communication**										
Using e-mail, texting or the Web to reach my health care provider	68/231 (29.4)	8	6/27 (22.2)	14	62/204 (30.4)	9	27/86 (31.4)	13	35/118 (29.7)	8
Making sure that all my diabetes care providers are working together for me	77/298 (25.8)	14	11/33 (33.3)	6	66/265 (24.9)	16	28/102 (27.5)	17	38/163 (23.3)	12
Making choices about diabetes medicine and other treatments that I think are best for me	76/301 (25.2)	16	5/31 (16.1)	18 (tie)	71/270 (26.3)	14	33/104 (31.7)	12	38/166 (22.9)	13
Feeling that my doctor or other health care providers respect,	69/314 (22.	17	9/33 (27.3)	9	60/281 (21.4	19	30/105 (28.6	16	30/176 (17.0	21

understand, and listen to me	0)				)		)		)	
Working with my doctor to set personal goals for my treatment	66/309 (21.4)	18	5/31 (16.1)	18 (tie)	61/278 (21.9)	17	27/105 (25.7)	18 (tie)	34/173 (19.7)	18
Talking with my health care provider about the pros and cons of each choice for my treatment	61/311 (19.6)	20	5/32 (15.6)	22	56/279 (20.1)	20	24/105 (22.9)	20	32/174 (18.4)	19
Getting a response from my health care provider in a timely manner	58/314 (18.5)	21	7/33 (21.2)	16 (tie)	51/281 (18.1)	21	27/105 (25.7)	18 (tie)	24/176 (13.6)	24
Understanding current and future health risks of my diabetes	52/314 (16.6)	23	2/32 (6.3)	26	50/282 (17.7)	22	19/107 (17.8)	24	31/175 (17.7)	20
Getting easy to understand instructions about taking care of my diabetes	65/309 (21.0)	19	5/31 (16.1)	18 (tie)	60/278 (21.6)	18	24/106 (22.6)	21	36/172 (20.9)	15
**Medications**										
Testing my blood sugars	85/308 (27.6)	11	8/33 (24.2)	11	77/275 (28.0)	10	35/107 (32.7)	11	42/168 (25.0)	10
Managing side effects of interactions between my medications	76/275 (27.6)	10	9/24 (37.5)	5	67/251 (26.7)	13	30/101 (29.7)	15	37/150 (24.7)	11
Using alternative medicine (natural herbs, acupuncture, meditation, etc.)	43/163 (26.4)	12	3/19 (15.8)	21	40/144 (27.8)	11	21/52 (40.4)	8	19/92 (20.7)	16
Taking insulin	23/163 (14.1)	26	2/33 (6.1)	27	NA[b]	NA	14/107 (13.1)	27	NA	NA
Taking diabetes medications (other than insulin) as prescribed	16/256 (6.3)	28	0/12 (0.0)	29	16/244 (6.6)	27	8/90 (8.9)	28	8/154 (5.2)	27
Taking medications for cholesterol or blood pressure as prescribed	13/272 (4.8)	29	1/21 (4.8)	28	12/251 (4.8)	28	8/99 (8.1)	29	4/1512 (2.6)	28
**Diet and Exercise**										
Getting enough physical activity	211/319 (66.1)	1	21/33 (63.6)	1	190/286 (66.4)	1	76/107 (71.0)	1	114/179 (63.7)	1 (tie)
Managing my weight	204/319	2	15/33	3 (tie)	189/286	2	75/107	2	114/179	1

	(64.0)		(45.5)		(66.1)		(70.1)		(63.7)	(tie)
Managing stress	178/317(56.2)	3	15/33 (45.5)	3 (tie)	163/284 (57.4)	3	64/107 (59.8)	3	99/177 (55.9)	3
Eating a healthy diet	161/319 (50.5)	4	10/33 (30.3)	8	151/286 (52.8)	4	56/107 (52.3)	4	95/179 (53.1)	4
**Relationships with Others**										
Trying not to be a burden to others	112/295 (38.0)	5	15/31 (48.4)	2	97/264 (36.7)	6	46/99 (46.5)	5	51/165 (30.9)	7
Getting enough support from my family and friends	117/317 (37.4)	6	7/32 (21.9)	15	110/281 (39.1)	5	44/106 (41.5)	7	66/175 (37.7)	5
Diabetes interfering with my work	51/194 (26.3(	13	7/30 (23.3)	12	44/164 (26.8)	12	22/63 (34.9)	9	22/101 (21.8)	14
Diabetes interfering with my social activities with family, friends, neighbors or groups	71/281 (25.3)	15	7/31 (22.6)	13	64/250 (25.6)	15	32/95 (33.7)	10	32/155 (20.6)	17

[a] Rank = Within each column, “1” is the domain with the highest percentage of participants responding “very difficult” or “somewhat difficult” and “29” is the domain with the lowest. “Tie” indicates that two domains had the same percentage of participants responding “very difficult” or somewhat difficult.”
[b] NA = not applicable.
