# Supplementary material for: An Innovative Approach to Informing Research: Gathering Perspectives on Diabetes Care Challenges From an Online Patient Community
Source: Interact J Med Res. 2015 Jun 30;4(2):e13. doi: 10.2196/ijmr.3856 (PMC4526969; doi:10.2196/ijmr.3856)
Supplement: Multimedia Appendix 3 [file ijmr_v4i2e13_app3.pdf]

Multimedia Appendix 3. Summary of responses to: "Thinking about when you were first told you had diabetes, what was or would have been most helpful for you to know about your diabetes at that time?", N=299[a]

Themes	n	Illustrative Quotes
**Lifestyle Education**	90	
• Education in diet, carbohydrate consumption, and food preparation	49	"I believe that if I had had more information about carb counting ten years ago that I would have been better off health wise with the diabetes than I am now. I did not receive dieting information until just recently."<br>"…a special session with someone who would discuss my lifestyle with me and then work with me to try to figure out how to best get exercise into my schedule, how to adjust my eating habits to eat healthier."<br>"A course on what to eat and how much... Even though I really like my doctor, all he did was tell me to research it on the internet. … His guide on what to eat – 'a small glass of orange juice or a small baked potato'...[needed something] other than that just 'eat healthy'."
• Clear, realistic assessment of lifestyle goals to work on (diet, exercise, smoking, etc)	16	
• Education and support in weight loss (i.e. gyms, classes, bariatric surgery)	15	
• Education on importance of active lifestyle and ongoing exercise routine	7	
• Encouragement to avoid stress and stay calm and relaxed	3	
**Shared Decision Making, Compassion, Reassurance, and Support**	87	
• Realistic discussion with provider about the seriousness of diabetes, that it requires diligent and daily self-care and treatment, and that it can be managed	28	"How extremely important it was and is to take care of yourself, the gravity of what this disease can do to your body and your life. How that if you do everything that you are supposed to do and more that you can feel good and live a full life…I should have been told that if I did everything that I do now I could have put off dealing with a lot of the problems that diabetes can cause, but I wasn't told those things."<br>"I wish [my doctors] would have helped me create a diabetes support team and not just left me on my own to deal with it."<br>"I was just given pamphlets, syringes and medication. Also an appointment for the next education session. What was I supposed to do in the meantime. My blood sugar was 400 and I had no idea if I should worry about it or not."
• At time of diagnosis, support in dealing with denial or overwhelming fear of diabetes	16	
• Hope and reassurance that a person with diabetes progression can be slowed, possibly reversed, and that a person can live a normal and good life	16	
• Open, two way discussion with provider on all aspects of diabetes and treatment options	13	
• Credible, helpful resources: information on diabetes (books, web, literature) and also social support groups	10	
• Compassion from provider with no blaming or shaming of person trying to manage their diabetes	4	
**Education on the Disease of Diabetes**	86	
• Education in diabetes	39	"How it was affecting my body and what was

progression, long-term consequences, side effects, and symptoms of worsening		going to happen if I didn't do anything to take care of it.”<br><br>“More in-depth knowledge about how diabetes functions/dis-functions within, not just how to measure glucose levels.”<br><br>“I wasn't told very much... PCP just said my blood sugar was too high and I needed metformin. I thought to myself "what sugar in my blood, I don't have sugar in my blood." I literally thought about sugar crystals in my veins, I didn't know what diabetes was and I don't think he even called it that.”
• Education on what diabetes is, its causes, and contributing factors	20	
• Clear explanation of blood sugars, A1c levels, and insulin resistance	8	
• Education on diabetes interactions and impacts on comorbid conditions (depression, hypothyroidism, kidney disease, hypoglycemia)	9	
• Awareness and education about transitioning from prediabetes to diabetes	6	
• Awareness and education about pregnancy and diabetes, including gestational diabetes	4	
**Diabetes Care Management Education**	25	
• Education on the importance and reason for routinely self-monitoring blood glucose levels	13	You can’t be perfect trying to have good control. High blood sugars doesn’t mean you are not trying hard enough.”<br><br>“What the cost would be of not changing my diet, not changing my exercise routine, etc. I don't know how much of a difference it would have made, but it would have been nice to know that I could do something at that time.”
• Stronger emphasis/education that diabetes can be managed and treated well with diet, lifestyle, and medications	12	
**Care Access, Referrals, and Costs**	24	
• Timely access/referrals to specialists (i.e. endocrinologists), dieticians, and diabetes educators/classes at time of diagnosis and as part of ongoing care	17	“I was working at a good paying job at that time. It would have been helpful to understand how soon all the side effects of the disease would show up and the cost of treatment, medications, and doctor visits would cost.”<br><br>“Being able to meet one-to-one with a diabetes educator and a nutritionist/dietician. This would have allowed me to ask questions pertinent to my situation and lifestyle. As it was, I didn’t even get group meetings.”
• Timely access to medications and durable equipment (i.e. insulin pump)	4	
• Realistic appraisal of immediate and long-term diabetes financial impact (i.e. provider costs, medications, blood glucose monitors, test strips)	3	
**Education about Diabetes Medications**	23	
• Clear explanations about medication side-effects (e.g. digestive, impotence, weight gain) and alternative medication	10	“That insulin makes you gain weight.”<br><br>“Better information about when to take my

options		medication. For example, I didn't know until about 2 years ago that I should take my metformin and glyburide at meal time, not just whenever I felt like it."
• Understanding of how medications are processed by the body, including the time at which they are taken	8	
• Education of non-pharmaceutical alternatives or providers	5	
**Other Comments**	**n**	**Illustrative Quotes**
**Received Good Education and Support / Not sure**		
• Have good understanding of disease, received good provider education and support, or have done enough research on their own	45	"I was given lots of information by my doctor & she sent me to a diabetes educator for help with diet. This was very helpful because getting the diagnosis of diabetes is overwhelming." "Nothing. I come from a long line of diabetics."
• Nothing, not sure at this time, diagnosis too long ago	26	
**Frustrations and Complaints**		
• Misdiagnoses or late diagnosis followed by poor provider communication and support at time of diagnosis and post-diagnosis	34	"First, my doctor thinks he identified me as having Type II diabetes in 2007 but he didn't tell me then. … According to my notes on medical visits and tests, it wasn't until 2009 that I was given a prescription to control a "Pre-Diabetes" condition. I was never actually told when it became Diabetes." "I have neuropathy thanks to [the hospital] never bothering back in 95 to tell me when I was going to their clinic that I had high blood sugar along with the high blood pressure they did tell me about. I started having numbness in my feet around that time and no one ever said to me anything…"
• Knowledge and understanding about diabetes was self-taught	10	
• Setting and circumstances of diagnosis (i.e., emergency room, hospital) contributes to shock for both patient and family and makes it difficult to absorb any information about the disease at that time.	3	

[a] Because an individuals' response could reflect multiple themes, the n's for the themes is greater than the number of respondents (N). The n's for the summary themes are the sum of the n's of the individual themes within that category.
