# Supplementary material for: An Innovative Approach to Informing Research: Gathering Perspectives on Diabetes Care Challenges From an Online Patient Community
Source: Interact J Med Res. 2015 Jun 30;4(2):e13. doi: 10.2196/ijmr.3856 (PMC4526969; doi:10.2196/ijmr.3856)
Supplement: Multimedia Appendix 4 [file ijmr_v4i2e13_app4.pdf]

Multimedia Appendix 4. Summary of responses to: "Thinking about 3-5 years in to the future from now, what do you feel will be important to learn or know about your diabetes? Why?" N=283[a]

Themes	n	Illustrative Quotes
**Understand Disease Progression**	120	
• Progression and impact on overall health and aging	60	"How the disease changes over time and the impacts. IT WOULD BE NICE to have a general TIMELINE of the disease progression to consult. Had the hardest time establishing that."<br><br>"How long does it take for organ failure if your blood sugar is out of control?"
• Progression and impact on specific organ systems (i.e. eyes, kidneys, heart, depression, neuropathy, etc.)	60	
**Long-term Control, Treatment, and Care Management Strategies**	106	
• How to effectively maintain and manage blood sugars over the years	54	"To survive this dreadful disease. I want to see my grandchildren. What else can go wrong? I thought I knew the secondary complications....But there are many more I am just learning about. How can this be? I am an educated woman....I have managed this disease (and successfully) for MOST of my life. I have recently started "falling apart". I know tight control is the answer....But I am so frustrated, and unable to get my numbers tight."<br><br>"REALLY aggressive education on avoiding complications of diabetes, because it's a lot easier to exercise if you are not missing parts of your feet, and easier to manage stress if you are not afraid of losing parts."<br><br>"I want to find a specialist that is prepared to talk to me about what my life is really like and what is possible for me to do knowing who I am."
• How to avoid or best manage medication use, especially insulin use	19	
• How to manage neuropathy, its associated pain, and prevent amputations	15	
• How to have an ongoing individualized, personalized, and integrated care management plan	10	
• How to continue to care for diabetes and know as much as possible about it over the years	8	
**Awareness of New Treatments and Technologies**	72	
• Current science, research, and technology for new ways to treat and manage diabetes	36	"The latest discoveries and treatments for diabetes. I want to be able to have better choices than my relatives."<br><br>"Because I cannot tell when my blood sugar is dropping until it reaches a dangerous level it would be extremely helpful if an implantable tool could be placed under the skin that would give me a general idea or range, say green for normal, yellow for dropping or beware and red for reaching dangerously low levels."
• Information on: new medications, new glucose management technologies, improved diet/lifestyle options	36	
**Long-term Lifestyle Management Strategies**	43	
• Knowing more about healthy eating, nutrition, and access to proper foods over the years	32	"Learn to eat fish. Learn to hate chocolate. Develop an allergy to potatoes and pasta. Learn

• Having an exercise routine tailored to a persons' lifestyle and physical limitations as years progress	11	to delight in squash, broccoli, and Brussel sprouts … without butter."
**Managing Future Costs**	40	
• How to manage and afford ongoing costs of medications, test supplies, foods, exercise fees, etc.	20	If I will still be able to control without insulin. Don't know if I can afford insulin." "… what can I do to help manage costs of diabetes without suffering physically." "My concern is mainly about costs, insurance and employment."
• How to manage and afford any increase in costs of medications, test supplies, foods, exercise fees, etc.	20	
**Reverse or Cure Diabetes**	37	
• Strongly desire a cure be found	28	'I know pretty much all there is to know about diabetes... I wish there would be a cure." "That it's curable which, in most cases of Type 2, it is."
• Desire to know how to reverse diabetes, including use of bariatric surgery	9	
**Managing Overall Health and Well-being**	23	
• How to live longer, healthier, happier, and better with diabetes	16	"I would like to know if neuropathy can be reversed. I am so sick & tired of being in pain 27/7/365. If I didn't hurt so much I would like to do new things & go places even if I can't see very well anymore." "I WANT TO LIVE LONGER AND ENJOY LIFE."
• How to accept living with diabetes and the need for lifestyle changes	7	
**Other Comments**	**n**	**Illustrative Quotes**
**Uncertain/Not Sure or Doing Fine**		"Doubt I'll be alive in 3-5 years and if I am, I really doubt I'll have medical coverage of any kind."
• Nothing or uncertain of future needs at this time	26	
• Fatalistic: Not sure will be alive in 3-5 years due to age, complications, and other serious health issues	12	
• Know what to expect and do to manage diabetes	11	

[a] Because an individuals' response could reflect multiple themes, the n's for the themes is greater than the number of respondents (N). The n's for the summary themes are the sum of the n's of the individual themes within that category.
