# Supplementary material for: An Innovative Approach to Informing Research: Gathering Perspectives on Diabetes Care Challenges From an Online Patient Community
Source: Interact J Med Res. 2015 Jun 30;4(2):e13. doi: 10.2196/ijmr.3856 (PMC4526969; doi:10.2196/ijmr.3856)
Supplement: Multimedia Appendix 5 [file ijmr_v4i2e13_app5.pdf]

Multimedia Appendix 5. Summary of responses to: "Are there any other challenges about your diabetes care that you want us to know?", N=257[a]

Themes	n	Illustrative Quotes
**Managing Blood Glucose, Medications, and Lifestyle Routines**	38	
• Discouragement and frustration with the ups and downs of blood sugar management	11	"My diabetes is totally uncontrolled. It ranges from 79 to over 600. I never know what it will be and I'm afraid that it will kill me.' "When I did bring my blood sugar readings down. It didn't last very long. Only for a couple of months, even though I hadn't changed anything. It was discouraging."
• Challenging to maintaining regular medication schedule or appropriate diet and exercise routine	11	
• Frustration in managing diabetes and having clear, easy, effective treatment and lifestyle protocols	8	
• Help and alternatives to better manage medication side effects like weight gain and digestive issues	8	
**Maintaining Healthy Lifestyle**	35	
• Eating healthy (i.e. low glycemic diet) is too difficult	12	"Eating healthy requires more food shopping and cooking that I really want to do at this point in life (age 67, and getting tired of the kitchen). This presents a constant challenge. I eat large, main course salads for several months and then get so sick of them that we do no more for a long time.' "Trying to eat what I'm supposed to eat for my health (diabetes) is difficult because if I eat correctly it is expensive. It's like you get penalized for trying to eat right. So I don't always get the kind of groceries I need."
• Challenge of being overweight and trying to lost weight	9	
• Inability to exercise due to other health issues, lack of access, and costs	9	
• Inability to eat healthy due to other health conditions, access and costs of recommended foods.	5	
**Care Access and Costs**	27	
• Difficulties with current and future costs / financial barriers related to care (medications, test strips, meters, etc.)	21	"Why is managing something that is so common so difficult? The price of the strips is ridiculous. If I didn't have insurance that covered it I would seriously be in some bad shape. I don't know what's available for people of lower incomes, but if I had diabetes and I was poor, I'd be concerned." "Taking care of the expenses: the expense of medicine, the expense of doctor visits, the expense of being away from work. All of these things, taken individually, are only bad to a point. Taken together, however, makes the cost prohibitive.'
• Lack of easy and efficient access to medications and testing supplies	6	
**Stress and Limitations of Diabetes on Daily Life**	26	

• Managing diabetes requires constant effort and has a big and often negative influence on daily life	9	“You just get so tired of all the things you have to do day after day after day. I am tired of it.’<br><br>‘People that don't have diabetes just don't realize how hard it is to deal with an illness like this. How it can interfere with your job and people will tempt me with sweets and stuff I don't want because then I worry about my blood sugar going up. “<br><br>“I know it can affect my eyes, my kidneys and I could face amputation. All of these things do terrify me, but it still doesn't scare me into thinking I must eat the right things, avoid eating bad things, exercise, lose weight. I do take cancer seriously (I've had breast cancer) and did everything the doctors said including lumpectomy, radiation, taking Tamoxifen. Somehow diabetes doesn't seem real.”
• Daily and yearly management is stressful, tiresome, and difficult to maintain motivation for appropriate care over time.	9	
• Diabetes limits social life making it difficult to engage in and enjoy activities	8	
**Managing Comorbid Conditions**	24	
• Other health conditions (i.e. cancer, fibromyalgia) may take higher priority and are difficult to manage due to diabetes	14	“I am worried that since I don't/can't exercise, it will make everything worse, especially my heart and my feet! But I have other issues, extreme lower back pain, and am unable to walk more than a minute or two. Any ideas?”<br><br>‘… taking care of my diabetes and then taking care of my Fibromylagia, Rheumatoid Arthritis, and Neuropathy, COPD, IBS, and ruptured discs in my neck and lower back.”
• Complications related to diabetes (i.e. neuropathy) are difficult and stressful to manage	10	
**Long-term Consequences of Diabetes**	20	
• Fear of future diabetes complications (death, organ failure, eyes, neuropathy, kidneys) and ability to prevent their development	15	“As I age I begin to think of the hand-off that is eventually inevitable, where I must continue my care through the assistance of another person, mainly my wife. Not sure how it will work out. As I age my memory is certainly affected and forgetting shots is a distinct possibility. Makes me a bit nervous.”
• Fear of self-managing diabetes when getting older (i.e. poor memory) and extra burden on family and friends	5	
**Provider Access and Support**	16	
• Lack of providers or access to providers/programs to help with diet and carbohydrate management	7	‘I think it would be beneficial to have access to a diabetes educator on more than just the occasion when you're first diagnosed and becoming insulin-dependent.’<br><br>“Keeping in communications with my diabetic team and them keeping in communication with each other.”
• Difficult to find a “good” doctor to work with and who will educate and coordinate care	5	
• Not enough ongoing provider support and education for long-term management of diabetes	4	

after the initial diagnosis		
**Lack of Social Support**	15	
• Feelings of depression, isolation, and sense of "not good enough"	8	"The hardest thing is the support from my family, especially my wife. She sees things her way and is not willing to change her views it seems when it comes to diabetes and me"<br><br>"I have never been diagnosed with depression, but sometimes it is a challenge to go and do some of the things I liked to do because of the fear of not being good enough anymore."
• Experiencing negative influences from caregivers and social support system such as: poor advice, lack of understanding about diabetes, or encouraging poor behaviors.	7	
**Lack of Up-to-Date Treatments and Cure**	11	
• Concern that most up-to-date diabetes management (i.e. medications, dietary recommendations) found in the literature or being used in other countries is not offered by providers	7	"Why are so few people studying cures? There are claimed cures in Europe and Asia, why is the US not studying them to see if they are true? Why is the surgical procedure not being studied more?"<br><br>"I would like to find a way to cure it as opposed to sustaining it by taking medication."
• Concern about finding a cure / lack of any cure	4	
**Other Comments**	**n**	**Illustrative Quotes**
**Uncertain/Not Sure or Doing Fine**		"No-this survey covered pretty much everything."
• Nothing or not sure at this time	112	
• Feel fine with care and glucose control at the moment	6	

[a] Because an individuals' response could reflect multiple themes, the n's for the themes is greater than the number of respondents (N). The n's for the summary themes are the sum of the n's of the individual themes within that category.
